# Supplementary material for: Short-term solar energy forecasting: Integrated computational intelligence of LSTMs and GRU
Source: PLoS One. 2023 Oct 4;18(10):e0285410. doi: 10.1371/journal.pone.0285410 (PMC10550187; doi:10.1371/journal.pone.0285410)
Supplement: Attachment — Submitted filename: review comments PONE-D-22-24267_R1_reviewer.pdf [file pone.0285410.s002.pdf]

TITLE: Short-term solar energy forecasting: Integrated computational intelligence of LSTMs and GRU

Manuscript ID : PONE-D-22-24267_R1_reviewer

Type of manuscript : Original Article

Summary

In this paper, the authors carry out a comparative analysis of the performance of using Lasso regression, ridge regression, ElasticNet regression, and Support Vector Regression, as well as deep learning models for time series analysis including long short-term memory (LSTM), bidirectional LSTM (Bi-LSTM), and gated recurrent unit (GRU) along with their variants for estimating solar energy generation for every five-minute interval on Amherst weather power station.

I found this paper interesting to read and is an important paper in renewable energy modelling and forecasting. However, the manuscript requires some improvements. Some general comments are:

General Comments

- In the "Abstract", the authors should briefly present and justify the novelty of their approach in contrast to the existing body of knowledge.
- Provide a list of abbreviations used in the paper.
- There are some typos and grammatical errors in the manuscript.
- Insert full stops at the end of the figure and table captions.
- At the end of every equation which is followed by "where", insert a comma. For example: $y = x + \beta x,$ (1)
  where β is a constant.
- It is usually not recommended to use MAPE and R-squared as evaluation metrics when working with renewable energies such as solar power. The authors are advised to read the following paper. Hong et al.: Energy Forecasting: A Review and Outlook https://doi.org/10.1109/OAJPE.2020.3029979 See for example the section on Common Issues.
- I suggest that the authors combine the forecasts from the prediction models. See for example Wang X., Hyndman R.J, Li F. and Kang Y. (2022). Forecast combinations: an over 50-year review. arXiv:2205.04216v1 and Mpfumali et al. (2019) http://dx.doi.org/10.3390/en12183569, among others. You may also want to provide the prediction intervals (PIs) for your best forecasting model. This is important as PIs capture the uncertainty surrounding the forecasts.
- Usually the three shrinkage methods Lasso, ridge and elaticnet are used for variable selection after which more advanced methods are then used for forecasting. I suggest that the authors compare the predictive capabilities of SVR, LSTM, Bi-LSTM and GRU on covariates selected by the three shrinkage methods. But should clearly show their contribution to this study.
- In the "Discussion" section the authors should present their findings and their main implications, also highlight the current limitations of their study and briefly mention

some precise directions that they intend to follow in their future research work. Can the authors mention how much of their research is being influenced by the data they used or to which extent the methodology used within the developed research can be easily applied to other situations when the datasets differ? In this way, the authors could highlight the generalisation capability of their approach to be able to justify a wider contribution that has been brought to the current state of the art.

- In the "Conclusion" section authors should avoid simply summarizing the aspects that they have already stated in the body of the manuscript. Instead, they should interpret their findings at a higher level of abstraction than in the previous sections of the manuscript. The authors should highlight whether, or to what extent they have managed to address the necessity identified within the "Introduction" section (the identified gap). The authors should avoid restating everything they did once again, but instead, they should emphasize what their findings mean to the readers, therefore making the "Conclusions" section interesting and memorable to them. The authors should not restate what they have done or what the article does, they should focus instead on what they have discovered and most importantly on what their findings mean.
