# Supplementary material for: Short-term solar energy forecasting: Integrated computational intelligence of LSTMs and GRU
Source: PLoS One. 2023 Oct 4;18(10):e0285410. doi: 10.1371/journal.pone.0285410 (PMC10550187; doi:10.1371/journal.pone.0285410)
Supplement: Attachment — Submitted filename: Response to Reviewers RNN.docx [file pone.0285410.s001.docx]

**Response to the comments on the submitted manuscript ID: PONE-D-22-24267**

**Paper ID:** PONE-D-22-24267

**Title:** Short- Short term solar energy forecasting: Integrated computational intelligence of LSTMs and GRU

**Authors:** Aneela Zameer, Fatima Jaffar, Farah Shahid, Muhammad Muneeb, Rizwan Khan, Rubina Nasir

The authors would like to thank the anonymous reviewers and editor for their valuable, detailed and constructive comments that allow us to further improve the quality of the paper. All the comments and suggestions are carefully addressed in this document and the manuscript is accordingly revised. In this response to the editor’s comments, the journal requirements are shown in italic purple font and the authors' response in green font.

The red color font of the text is used for ease in the identification of the changes/references in the body of the revised manuscript.

***Reviewer 1 Comment 1***

*The topic of the manuscript is interesting and significant. However, the Introduction is very poor, and the content looks too disorderly. The introduction is the comprehensive reference and deep analysis, instead of the simple list of documents. New ideas and theories (or application of those to existing problems) should be presented if they are comprehensively compared against state-of-the-art methods for many different problems and for a wide range of empirical instances in order to be convincing. Otherwise, there is little gain for the scientific community for yet another analytic method. I suggest that the Introduction should be improved and it should be also more artistic.*

**Author’s Response**: Thank you for your valuable comment! We have updated the introduction to explain the novelty of the manuscript in section 2. The new theories related to this study have been modified in the revised manuscript in introduction section 1.

***Reviewer 1 Comment 2***

*The motivation of using the proposed method is not given in this work. The novelty of the approach should be highlighted.*

**Author’s Response**:

Many thanks for your encouraging and valuable comments. The authors have tried their level best to improve the revised manuscript as per your worthy comments and suggestions. The presented work and methodology have been introduced first time with the novelty of time series data analysis through time series regression model GRU and LSTM used multi-location features to forecast t+1, t+3, t+6, t+12, and t+24 multistep ahead UMass dataset of solar power and which has also been clearly mentioned in the highlights of the introduction section, abstract and conclusion along with proof in the form of results, comparison with existing techniques. The proposed technique has been employed on Davis Meteorological station in Amherst, Massachusetts dataset. It has the potential to be implemented for any dataset in this domain. The novelty of the manuscript is clearly stated in the revised manuscript. For example, the novelty is explained in the last two paragraphs of the Introduction section (Page 8) explaining that the models are configured in a novel way and such deep learning models have not previously been applied to a region-wise dataset.

***Reviewer 1 Comment 3***

*Please give the application of solar energy forecasting. And the logicality and artistic quality should be considered to present the high quality of this paper. Therefore, the presentation and the statement should be improved strongly.*

**Author’s Response**:

Many thanks for your encouraging and positive comments. Authors have tried their level best to incorporate all the suggestions by esteemed reviewers to enhance the quality of their manuscript. Now Authors have added the application of solar energy forecasting in the introduction in the revised manuscript.

***Reviewer 1 Comment 1***

***Reviewer 2 General Comment***

*In this paper, long short-term memory, bidirectional LSTM, and gated recurrent unit have been used for estimating solar energy generation for every five-minute interval. My comments are:*

**Author’s Response**: Thank you for your valuable comment. We admire your professionalism and attentiveness. The authors have carefully revised the whole manuscript and more focused analysis on the comparison with other techniques to improve the quality of our revised manuscript.

***Reviewer 2 Comment 1***

*The proposed method consists of some well-known methods. The proposed method novelty should be clarified.*

**Author’s Response**: Agreed. Thank you for your precious and positive reviewing comments to improve the quality of our manuscript. The authors have carefully checked and improved the proposed methodology in the revised manuscript. A brief introduction related to machine learning models useful for regression problem is presented first in section 2.3 and then time series regression models related to recurrent neural networks (LSTM, GRU) is explained in section 2.3.2. Along with this, the presented work and methodology have been introduced first time with the novelty of GRU and LSTM using multi-location features to forecast t+1, t+3, t+6, t+12, and t+24 multistep ahead solar energy forecasting which has also been mentioned in the highlights of the introduction section, abstract and conclusion along with proof in the form of results, comparison with existing techniques.

***Reviewer 2 Comment 2***

*Why the proposed model is useful for solar energy generation forecasting? Many accurate methods based on deep learning has been presented recently.*

**Author’s Response**: Authors have revised the Introduction section carefully keeping your suggestions at hand. The problem statement and motivation have been revised for a better understanding of readers. Salient features of the proposed methodology and novelty have also been clearly stated in the introduction from Page 2 to Page 5 of the revised manuscript.

***Reviewer 2 Comment 3***

*Have the weather conditions amount been forecasted for next hours? Which method? What was prediction error?*

**Author’s Response**: Many thanks for your valuable remarks. Our dataset is related to the next five minutes and LSTM has the capability to learn the previous time steps and predict the next time steps. The experimental results section 2 (Page 16) is revised to provide insight into the model workings.

***Reviewer 2 Comment 4***

*Which optimization method has been used for the hyper parameters optimal selection?*

**Author’s Response**: Many thanks for your valuable comments. The important hyper-parameters that is the number of hidden neurons, the optimizer, and the number of lags (look back) essential to be decided. Parameter optimization plays a very important role to achieve higher accuracy, better efficiency, and good convergence. We executed the Grad student descent/(babysitting AKA) trial and error rule to optimize each hyperparameter as listed in Table 1, keeping the rest constant, and then selected the best parameter values as a trade-off between accuracy and time consumption. The experimental results for parameter optimization used a different number of Lags, which is achieved by computing the best score of the validation set, based on the score number of lags selected. Figure 11 (Page 27) presented the Learning curves of LSTM-V, Bi-LSTM, and GRU-II techniques with the best performance in terms of selecting the data records for forecasting and described as well as in Table 1-2 (Section 3, value selection for the Lag parameter and controlled parameter setting of LSTM, GRU, and BI-LSTM models, Page 19) and updated paragraph in subsection 2 of the revised manuscript.

Finally, the authors thank again the Editor in Chief, Associate Editor, Editorial Staff and the anonymous Reviewers for their support and efforts to improve the quality of the manuscript.

With best Regards

Aneela Zameer Jaafery

Corresponding author

Professor, DCIS

PIEAS, Islamabad, Pakistan
