# Supplementary material for: Short-term solar energy forecasting: Integrated computational intelligence of LSTMs and GRU
Source: PLoS One. 2023 Oct 4;18(10):e0285410. doi: 10.1371/journal.pone.0285410 (PMC10550187; doi:10.1371/journal.pone.0285410)
Supplement: Attachment — Submitted filename: Response to reviewers and Editor.docx [file pone.0285410.s003.docx]

**Response to reviewers**

Manuscript No.: PONE-D-22-24267R1

**Title:** **Short-term solar energy forecasting: Integrated computational intelligence of LSTMs and GRU**

**Authors:** Aneela Zameer^1#^, Fatima Jaffar^2^, Farah Shahid^3^, Muhammad Muneeb^4^, Rizwan Khan^5^, Rubina Nasir

The authors would like to thank the anonymous reviewers and editor for their valuable, detailed, and constructive comments that allow us to further improve the quality of the paper. All the comments and suggestions are carefully addressed in this document and the manuscript is accordingly revised. In this response to the editor’s comments, the journal requirements are shown in italic purple font, and authors' responses in green font.

**Reviewer #1 General Comments**

*The abstract should not contain abbreviation. The most core framework, contribution and results should be presented in the abstract. Please gives it within one paragraph.*

**Author’s Response**: Many thanks for your encouraging and positive comments. Authors have tried their level best to incorporate all the suggestions by esteemed reviewers to enhance the quality of their manuscript for the reputed Journal. The author has removed the abbreviations and modified the abstract according to the journal format.

*1. Whether the 0 value and NaN value exist in the dataset. So how do you handle it.*

**Author’s Response**: Thank you very much for your precious and positive reviewing comments. The authors have added the figure 4 which shows the feature visualization. Dataset has no NAN values.

*2. Please check the format about you title in the manuscript. ‘2.3.3 Performance Measures’ should be given in topcase writing.*

**Author’s Response**: Agreed. Authors modified the 2.3.3 section of performance measures in uppercase writing as per reviewer suggestions.

*3. There exists many one paragraph, which means logicality and artistic quality should be improved.*

**Author’s Response**: Agreed. The authors have proofread the manuscript carefully and critically and improved the quality of the revised manuscript.

**Reviewer #3**

*I found this paper interesting to read and is an important paper in renewable energy modelling and forecasting.*

**Author’s Response**: Thank you for your encouraging comment!

**General Comments**

- *In the “Abstract”, the authors should briefly present and justify the novelty of their approach in contrast to the existing body of knowledge.*

**Author’s Response**: We have modified the abstract as per the reviewer’s suggestion.

- *Provide a list of abbreviations used in the paper.*

**Author’s Response**: We have added a list of abbreviations as per the reviewer’s suggestions.

- *There are some typos and grammatical errors in the manuscript.*

**Author’s Response**: Thanks for your comment! The authors have carefully reviewed the manuscript for typos.

- *Insert full stops at the end of the figure and table captions.*

**Author’s Response**: Done! The authors have added the full stops at the end of each figure and table caption.

- *At the end of every equation which is followed by “where”, insert a comma. For example: y=x+βx, (1), where β is a constant.*

**Author’s Response**: Agreed! We have modified the equations as per the reviewer’s suggestions.

- *It is usually not recommended to use MAPE and R-squared as evaluation metrics when working with renewable energies such as solar power. The authors are advised to read the following paper. Hong et al.: Energy Forecasting: A Review and Outlook https://doi.org/10.1109/OAJPE.2020.3029979 See for example the section on Common Issues.*

**Author’s Response**: Thanks for your comment! The authors have thoroughly read the suggested link to use the MAPE and R-Squared. “As the evaluation metrics are often inadequate. For instance, Mean Absolute Percentage Error is used for close to zero load prices or renewable power generation. Sometimes authors tend to pick the error measures in favor of their proposed method but hide the results from other error measures”. We mentioned fair results of performance measures MAPE for our dataset and showed the comparison of the higher values of MAPE for the traditional model and less error for the proposed methodology. The authors have carefully checked the results in the revised manuscript.

- *I suggest that the authors combine the forecasts from the prediction models. See for example Wang X., Hyndman R.J, Li F. and Kang Y. (2022). Forecast combinations: an over 50-year review. arXiv:2205.04216v1 and Mpfumali et al. (2019) http://dx.doi.org/10.3390/en12183569, among others. You may also want to provide the prediction intervals (PIs) for your best forecasting model. This is important as PIs capture the uncertainty surrounding the forecasts.*

**Author’s Response**: Thanks for your comment! Your comments are very valuable. Authors have carefully checked the mentioned paper links and read the strategy to combine the forecasts and predict the interval data over these predictions. Although, PI’s are important to improving the accuracy of solar forecasts. Authors have tried their best to incorporate the idea of reviewer within short time span, and performed the analysis on the predicted values by the best model.

Figure 18: Prediction intervals on test data of forecast values

The prediction interval for the test set at a 95% confidence level is [-0.00213071 0.44163999], and the result is displayed in Figure 18. It can be observed that the predicted values fall well within this interval. The RMSFE method to calculate the prediction interval is very similar to RMSE but calculations are made on unseen data.

- *Usually the three shrinkage methods Lasso, ridge and elaticnet are used for variable selection after which more advanced methods are then used for forecasting. I suggest that the authors compare the predictive capabilities of SVR, LSTM, Bi-LSTM and GRU on covariates selected by the three shrinkage methods. But should clearly show their contribution to this study.*

**Author’s Response**: Many thanks for your encouraging and valuable comments. The authors have tried their level best to improve the revised manuscript as per your worthy comments and suggestions. In this study, Author’s main contribution to use the time series analysis on the solar dataset and compare the proposed model with traditional models that can be used for energy forecasting. Each one of them has capabilities of feature selection and prediction. However, we have added a comparison of the suggested model with the conventional one. Machine learning methods Lasso, ridge, and elastic net are used for attribute selection and prediction.

- *In the “Discussion” section the authors should present their findings and their main implications, also highlight the current limitations of their study and briefly mention some precise directions that they intend to follow in their future research work. Can the authors mention how much of their research is being influenced by the data they used or to which extent the methodology used within the developed research can be easily applied to other situations when the datasets differ? In this way, the authors could highlight the generalisation capability of their approach to be able to justify a wider contribution that has been brought to the current state of the art.*

**Author’s Response**: The authors have tried their level best to improve the revised manuscript as per your worthy comments and suggestions. The presented work and methodology of recurrent neural networks have been introduced for solar energy are commonly employed. However, this model can be implemented on different datasets. The efficacy of the model can be checked through the results where unseen data give a remarkable performance over other models.

- *In the "Conclusion" section authors should avoid simply summarizing the aspects that they have already stated in the body of the manuscript. Instead, they should interpret their findings at a higher level of abstraction than in the previous sections of the manuscript. The authors should highlight whether, or to what extent they have managed to address the necessity identified within the "Introduction" section (the identified gap). The authors should avoid restating everything they did once again, but instead, they should emphasize what their findings mean to the readers, therefore making the "Conclusions" section interesting and memorable to them. The authors should not restate what they have done or what the article does, they should focus instead on what they have discovered and most importantly on what their findings mean.*

**Author’s Response**: Agreed. The authors have tried their level best to improve the revised manuscript as per your worthy comments and suggestions.

The authors have tried their best to incorporate all the requirements of the esteemed journal with full devotion and concentration. We thank the Editor in Chief and Associate Editor to provide us with an opportunity to improve the quality of our manuscript through their suggestions and recommendations. We pay gratitude to the editorial staff and anonymous reviewers for their support, encouraging comments, and efforts to improve the quality of the manuscript.

With best Regards

Aneela Zameer Jaafery

Corresponding author

Professor, DCIS

PIEAS, Islamabad, Pakistan
